# ID4 regulates transcriptional activity of wild type and mutant p53 via K373 acetylation

**DOI:** 10.18632/oncotarget.13701

**Published:** 2016-11-29

**Authors:** Derrick J Morton, Divya Patel, Jugal Joshi, Aisha Hunt, Ashley E Knowell, Jaideep Chaudhary

**Affiliations:** ^1^ Department of Biology, Center for Cancer Research and Therapeutics Development, Clark Atlanta University, Atlanta, GA 30314, USA; ^2^ Department of Bioengineering Sciences, South Carolina State University, Orangeburg, SC 29117, USA

**Keywords:** ID4, bHLH, p53, mutant-p53, tumor suppressor

## Abstract

Given that mutated p53 (50% of all human cancers) is over-expressed in many cancers, restoration of mutant p53 to its wild type biological function has been sought after as cancer therapy. The conformational flexibility has allowed to restore the normal biological function of mutant p53 by short peptides and small molecule compounds. Recently, studies have focused on physiological mechanisms such as acetylation of lysine residues to rescue the wild type activity of mutant p53. Using p53 null prostate cancer cell line we show that ID4 dependent acetylation promotes mutant p53 DNA-binding capabilities to its wild type consensus sequence, thus regulating p53-dependent target genes leading to subsequent cell cycle arrest and apoptosis. Specifically, by using wild type, mutant (P223L, V274F, R175H, R273H), acetylation mimics (K320Q and K373Q) and non-acetylation mimics (K320R and K373R) of p53, we identify that ID4 promotes acetylation of K373 and to a lesser extent K320, in turn restoring p53-dependent biological activities. Together, our data provides a molecular understanding of ID4 dependent acetylation that suggests a strategy of enhancing p53 acetylation at sites K373 and K320 that may serve as a viable mechanism of physiological restoration of mutant p53 to its wild type biological function.

## INTRODUCTION

The somatic p53 missense mutations observed in almost half of all human cancers is a critical step in the oncogenic process [1, 2]. The consequences of p53 mutations (gain-of-function, GOF) within a cell can have at least three types of outcomes [3–6]: 1) p53 mutations can abrogate the tumor suppressor function of the affected TP53 allele by reducing the overall capacity of the cell to mount a proper DNA damage response [6, 7], 2) the mutant p53 may exert dominant–negative effect over co-expressed wild type p53 by forming mixed tetramers that are incapable of DNA binding and transactivation [6, 8] and 3) the mutant p53 protein with activities of its own, can contribute to various aspects of tumor progression [6, 9, 10].

Mutant p53 can be classified into two groups: type I mutations, which affect amino acid residues directly involved in the DNA interaction (R248H, V274F, and R273H), and class II mutations involving residues responsible for stabilization of the three-dimensional structure of p53 [1, 11]. Structural mutants, includes the majority of p53 proteins found in human tumors, such as R175H, P223L, R249H, and R282H mutants, all of which destabilize p53 conformation and the p53 DNA-binding [1, 2].

Structurally, wild type p53 consists of unfolded regions with high tendency for aggregation [12, 13]Numerous studies of mutant p53 have been designed to explore whether the DNA binding capacity can be restored artificially by small molecules which can stabilize the interaction with DNA, by preventing mis-folding or aggregation, site-specific phosphorylation and c- terminal amino acid substitution that mimics wild type p53 [2, 14–16]. Theoretical modeling of p53 has also been central to understanding the DNA-binding capabilities of mutant p53, as well as conformation changes induced by protein-protein interactions [17, 18]. The binding of small molecules such as CP-31398 [19], ellipticine [20], MIRA-1 [21, 22], RITA [22], and PRIMA-1 [23] to mutant p53 proteins may induce wild type like conformational changes in the DNA binding domains of p53 mutant proteins, restoring sequence-specific p53 transcription [24]. In some cases, however, the mechanisms of activation is less understood, which provides a physiological basis of exploration into this phenomenon.

Studies have shown that the all p53 mutations are not functionally equivalent [9]. The mutations in the DNA-contacting residues of p53 have subtle effect on the folding of p53 protein as compared to the structural mutants [25]. The mutant p53 can also interact with transcription factors and recruited to respective binding sites of those factors on chromatin, and modulate their transcriptional output [26]. Incidentally, certain p53 mutants can activate similar genes known to be activated by wild type p53 and induce senescence or apoptosis in some cell types but not in others providing a physiologically relevant basis to study strategies, which can restore mutant p53 to wild type biological activity by non-artificial means.

We and others have shown that in the case of wild type and mutant forms of p53, the interaction with acetylases and subsequent acetylation of p53 itself is indispensable for DNA binding and transcriptional activity such as the ability of p53 to trigger cell cycle arrest or apoptosis [1, 12, 27, 28]. Our previous study provided evidence that ID4, a helix loop helix transcriptional regulator recruited CBP/p300 (acetyltransferase) to promote large macromolecular assembly of p53 that could result in its acetylation and increased biological activity [28].

Here, we demonstrate that ID4, can promote p53-dependent apoptosis and senescence in prostate cancer cells by specifically modifying the acetylation of p53, which increases its transcriptional activity and promotes the expression of pro-apoptotic and cell cycle regulatory genes. Furthermore, using acetylation mimics of mutant p53, we identify lysine 373 and to a lesser extent lysine 320 as critical ID4 dependent acetylation sites that aid in p53 biological activities. Together, the results presented herein suggest that ID4 dependent p53 acetylation can be used as model to re-activate mutant p53 to wild type biological activity.

## RESULTS

**Figure 1:**
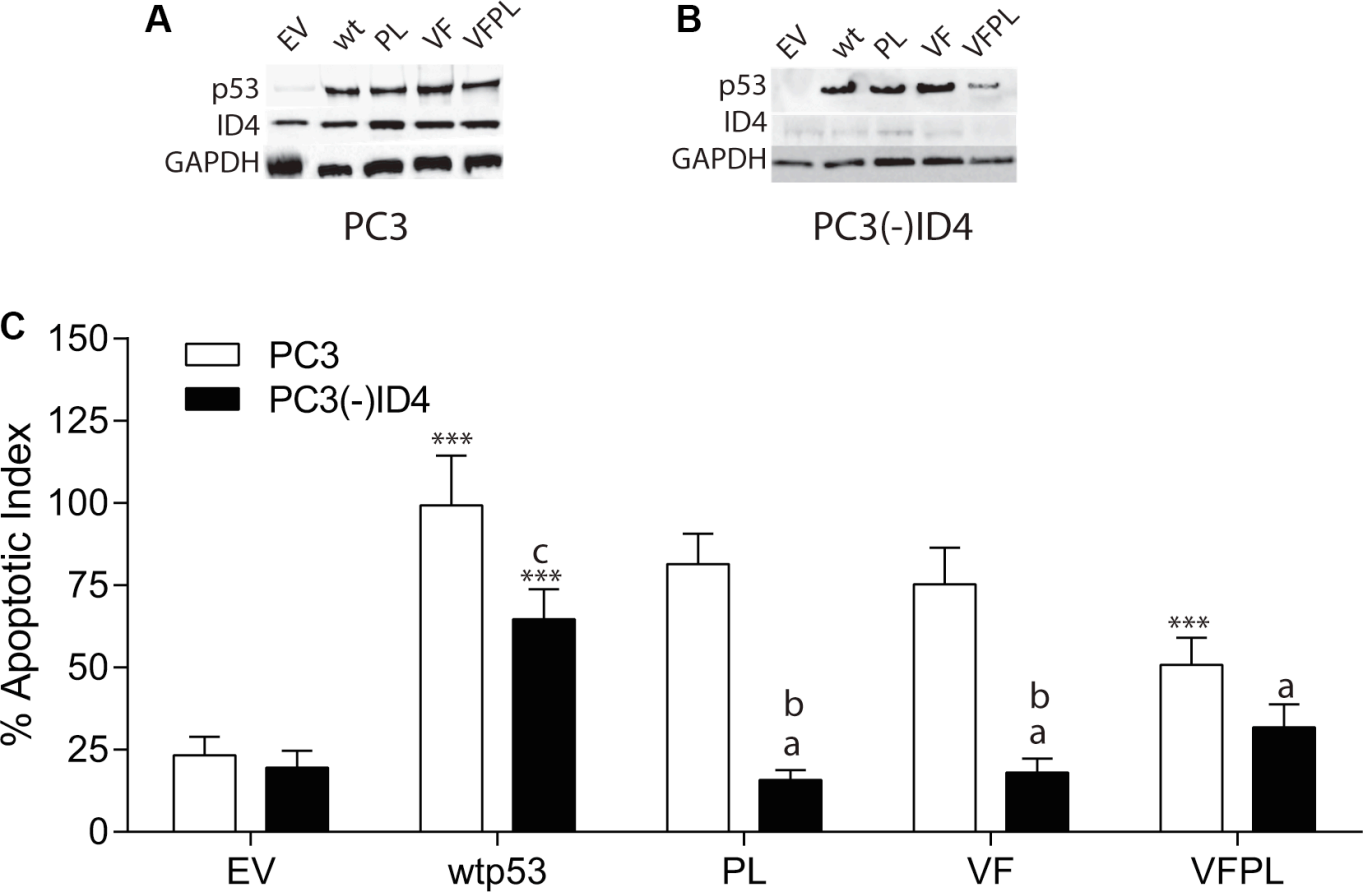
Stable knockdown of ID4 by retroviral shRNA in PC3 cells and expression of wild type and mutant p53 (**A**) Expression of ID4 and p53 in PC3 cells following stable transfection with wild type and mutant p53 plasmids. (**B**) Expression of ID4 and p53 in PC3 cells in which ID4 was stably silenced ((PC3(-)ID4) followed by stable expression wild and mutant p53 plasmids. (**C**) ID4 promotes p53-mediated apoptosis. Data represents apoptotic index calculated as percent of apoptosis as compared to apoptosis in PC3 cells transfected with wild type p53 set to 100%. A significant increase in apoptosis (***P < 0.001) in wild type and p53 mutants (p53 mutants: P223L and V274F) as compared to PC3 (EV); “a” (*P* < 0.001) in PC3 (–)ID4 with various p53 mutants as compared to PC3(-)ID4 cells transfected with wild type p53 and “b” (*P* < 0.001) and “c” (*P* < 0.05) is the statistical difference between PC3 and PC3(-)ID4 cells transfected with the same wild type or mutant p53 plasmid. Abbreviations: EV: Empty vector, wild type: Wild Type, PL: P223L, VF: V274F, VFPL: V274F+P223L.

The PCa cell line PC3 was used to investigate ID4-p53 cross-talk. Specifically, we focused on the role of ID4 in regulating wild type and mutant p53 dependent apoptosis, senescence and transcriptional activity. The PC3 cell line is null for p53 and has low endogenous levels of ID4 (Figure 1A). Wild type p53 as well as various mutant p53 plasmids (P223l: PL, V274F: VF, P223L+V274F: VFPL, R273H and R175H) were over-expressed (stable and/or transient) in the cells. Following stable transfection and antibiotic selection the clones stably expressing wild type or mutant p53 (Figure 1B and 1C) were expanded for subsequent studies.

Stable Silencing of ID4 and Expression of wild type and mutant P53 in PC3 cells: ID4 expression (Figure 1A) was undetectable or below detection limit in PC3 cells stably expressing the shRNA ID4 plasmid (PC3(-)ID4) (Figure 1B). As expected, p53 was not expressed in the PC3 cells transfected with empty vector (EV). The expression of wild type and mutant (PL, VF and VFPL) in PC3 and (PC3(-)ID4) suggested successful transfection and selection of the cell lines. The expression levels of the wild type and mutant p53 proteins in PC3 (Figure 1A) and PC3(-)ID4 (Figure 1B) was similar with the exception of VFPL mutant in PC3(-)ID4 (Figure 1B). The lower expression of VFPL mutant in PC3(-)ID4 could be due to multiple reasons e.g. destabilization of VFPL hetero-tetramer in the absence of ID4. The results discussed below should therefore be interpreted in context of the observation that the combined VFPL mutant expression is comparatively lower in PC3(-)ID4 cells compared to the wild type and PL and VF p53 mutant.

### ID4 promotes p53-mediated apoptosis

As expected a significant increase in apoptosis was observed in PC3+wild type p53 as compared to PC3 cells expressing the plasmid only control (EV) (Figure 1C, P < 0.001). The degree of apoptosis in PC3+wild type p53 cells was then used to normalize the apoptosis observed in response to various mutant forms of p53 in PC3 with or without ID4 (%apoptotic index) (Figure 1C). In PC3 cells, with the exception of VFPL which demonstrated a significant decrease in apoptosis (*P* < 0.001), the decrease in apoptosis of VF and PL was only marginal (75.3% and 81.4% respectively) as compared to wild type p53 (set to 100%). Thus the two mutant forms of p53 (VFPL) expressed together (as expressed in DU145 cells) were less effective in promoting apoptosis (50.8%) as compared to wild type p53 or VF and PL mutants expressed separately (Figure 1C). These results suggest that VF and PL mutants retain most of the biological activity of the wild type p53.

The apoptotic index of PC3(-)ID4 with wild type p53 (64%) was significantly lower as compared to PC3 cells with wild type p53 (100%, *P* < 0.05). Interestingly, a significantly lower apoptotic index was observed in PC3(-)ID4 cells expressing PL and VF mutants (PL: 15.7% and VF: 18.1%, *P* < 0.001) as compared to PC3(-)ID4 cells with wild type p53 (100%) and as compared to PC3 cells with PL and VF mutants (75.3% and 81.4% respectively) (Figure 1C).The percentage apoptotic index in the double VFPL mutant (31.8%, *P* < 0.05) was higher compared to the mutants expressed individually in PC3(-)ID4. The results suggested that loss of ID4 leads to a decrease in apoptosis overall but has a significantly stronger effect on the apoptosis induced by the two mutant alone (VF and PL) as compared to the two mutants together (VFPL). Thus, the results indicate that p53 promotes apoptosis in an ID4 dependent manner in PC3 cells.

### ID4 regulates expression of p53 targets genes

The expression of key downstream targets of p53 were used to investigate the mechanism by which wild type and mutant p53 regulates apoptosis in the presence or absence of ID4.

**Figure 2:**
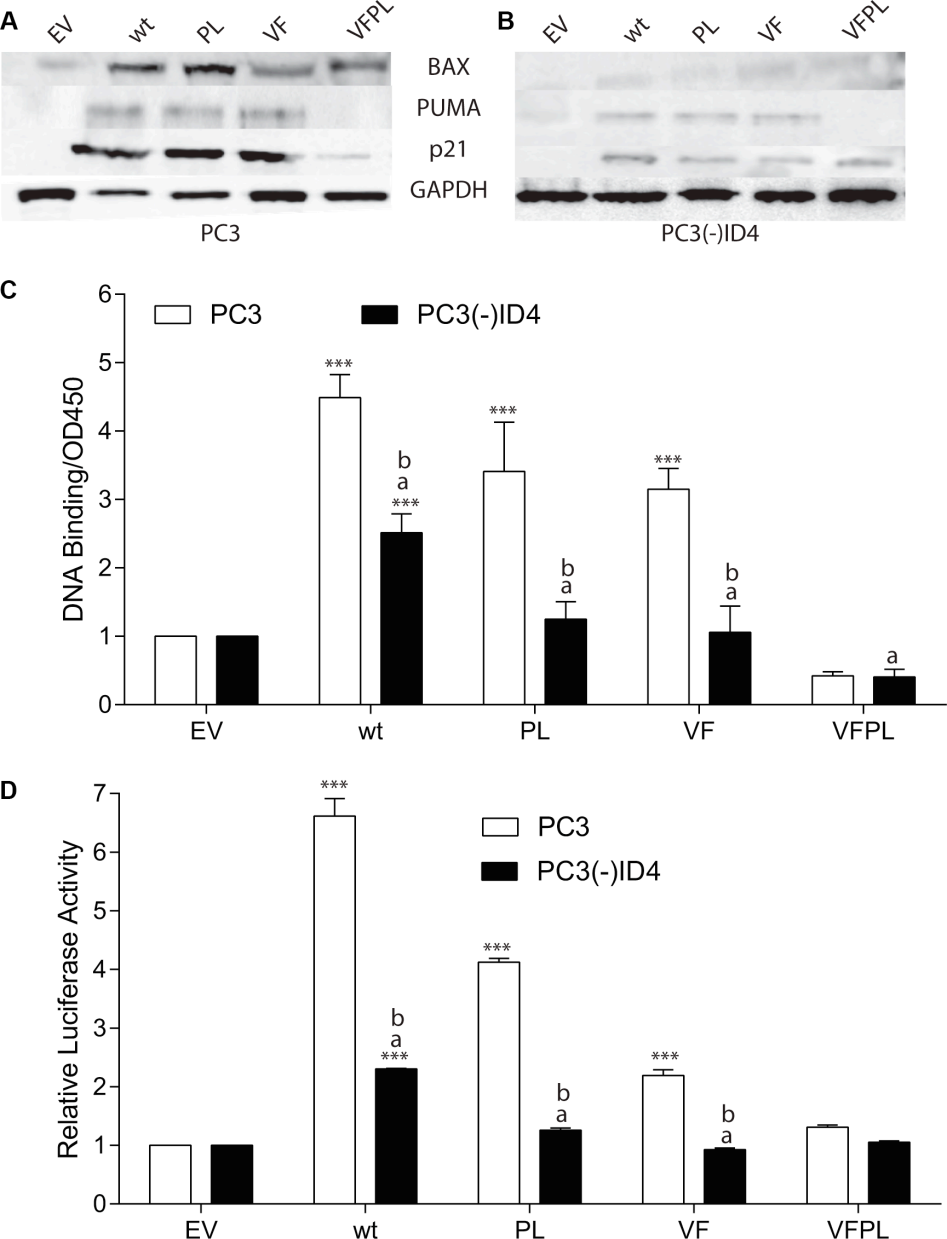
ID4 regulates expression of p53 targets genes (**A**) Protein expression of p21, BAX, and PUMA in PC3 and (**B**) PC3 (-) ID4 cells expressing Empty vector (EV), wild type (wild type), and mutant p53 (PL: P223L, VF: V274F, VFPL: V274F+P223L). Representative western blots of three different experiments are shown. (**C**) Quantitative p53 DNA binding in a sandwich ELISA. ***:*P* < 0.001 in wild type and p53 mutants as compared to PC3 (EV); “a” (*P* < 0.001) in PC3 (-)ID4 with various p53 mutants as compared to PC3(-)ID4 cells transfected with wild type p53 and “b” (*P* < 0.001) is the statistical difference between PC3 and PC3(-)ID4 cells transfected with the same wild type or mutant p53 plasmid.

The expression of cyclin dependent kinase inhibitor CDKN1A (p21), BAX and PUMA, which are well characterized p53 target genes increased in the presence of wild type and mutant p53 as compared to cells transfected with EV (Figure 2A) [29]. Interestingly, the expression of BAX between wild type p53 and PL mutant was essentially similar and higher than that observed in VF and VFPL. The expression profile of PUMA was also similar to that of BAX. The expression of p21 was similar between the wild type p53, PL and VF. The immunoblot analysis suggested that BAX, PUMA and p21 are expressed at a lower level in cells transfected with VFPL as compared to the PC3 cells transfected with these mutants alone. The expression level in VFPL correlates well with the extent of apoptosis in VFPL as compared to other p53 transfectants. The results suggested that similar to p53, the VF and PL mutants could be transcriptionally active.

Loss of ID4 resulted in a significant decrease in the expression of all three p53 target genes in PC3(-)ID4 cells transfected with either wild type or mutant p53. These results suggested that ID4 expression may be required to promote the activation of p53 downstream targets, irrespective of the p53 mutations (Figure 2B).

### ID4 restores mutant p53 DNA binding and transcriptional activity

An ID4 dependent gain in p53 downstream targets lead us to investigate whether wild type and mutant p53 in PC3 cells regain DNA binding and transcriptional activity in an ID4 dependent manner. Based on an *in vitro* p53 DNA binding activity assay (using p53 response element immobilized on a 96 well plate followed by detection with p53 specific antibody) using nuclear extracts, a significant increase in p53 DNA binding activity in the PC3+p53 (wild type and mutant p53) as compared the PC3(-)ID4 was observed (*p* < 0.001) (Figure 2C) with corresponding p53 wild type and mutant plasmids. The mutant VFPL p53 binding in PC(-)ID4 was significantly lowered as compared to wild type p53 and was comparable to EV. However, the DNA binding was also observed with VF and PL mutants in PC3 cells suggesting that these mutants are capable of DNA binding, possibly in the presence of ID4. No DNA binding was observed in the VFPL mutants which is consistent with the lack of its function (transactivation and apoptotic activity).

We next performed a functional assay to further confirm the p53 transcriptional activity in PC3 cells. Using a p53 response element (wild type-p53RE) luciferase reporter plasmid assay, the relative p53 luciferase activity decreased significantly in PC3(-)ID4+p53 (wild type and mutant) as compared to PC3+p53 (wild type and mutant) cells (Figure 2D). Surprisingly, mutant p53 (PL and VF) also demonstrated moderately high luciferase activity in PC3 cells. The mutant p53 luciferase plasmid (mt-p53RE) used as a negative control, as expected, did not result in luciferase activity (Data not shown). In context of PC3+wild type p53 used as a positive control, our results strongly suggested that mutant p53 gains DNA binding and transcriptional activity in the presence of ID4.

### ID4 promotes p53-mediated senescence

Having established that ID4 plays a critical role in modulating wild type and mutant p53 associated apoptosis, through the induction of a cell cycle checkpoint p21 and apoptosis (BAX and PUMA), we next investigated whether p53 also regulates cellular senescence in an ID4 dependent or independent manner. Senescence in normal cells is associated with a flattened, enlarged morphology with > 2-fold increase in cellular diameter compared to non-senescent cells [5].

**Figure 3:**
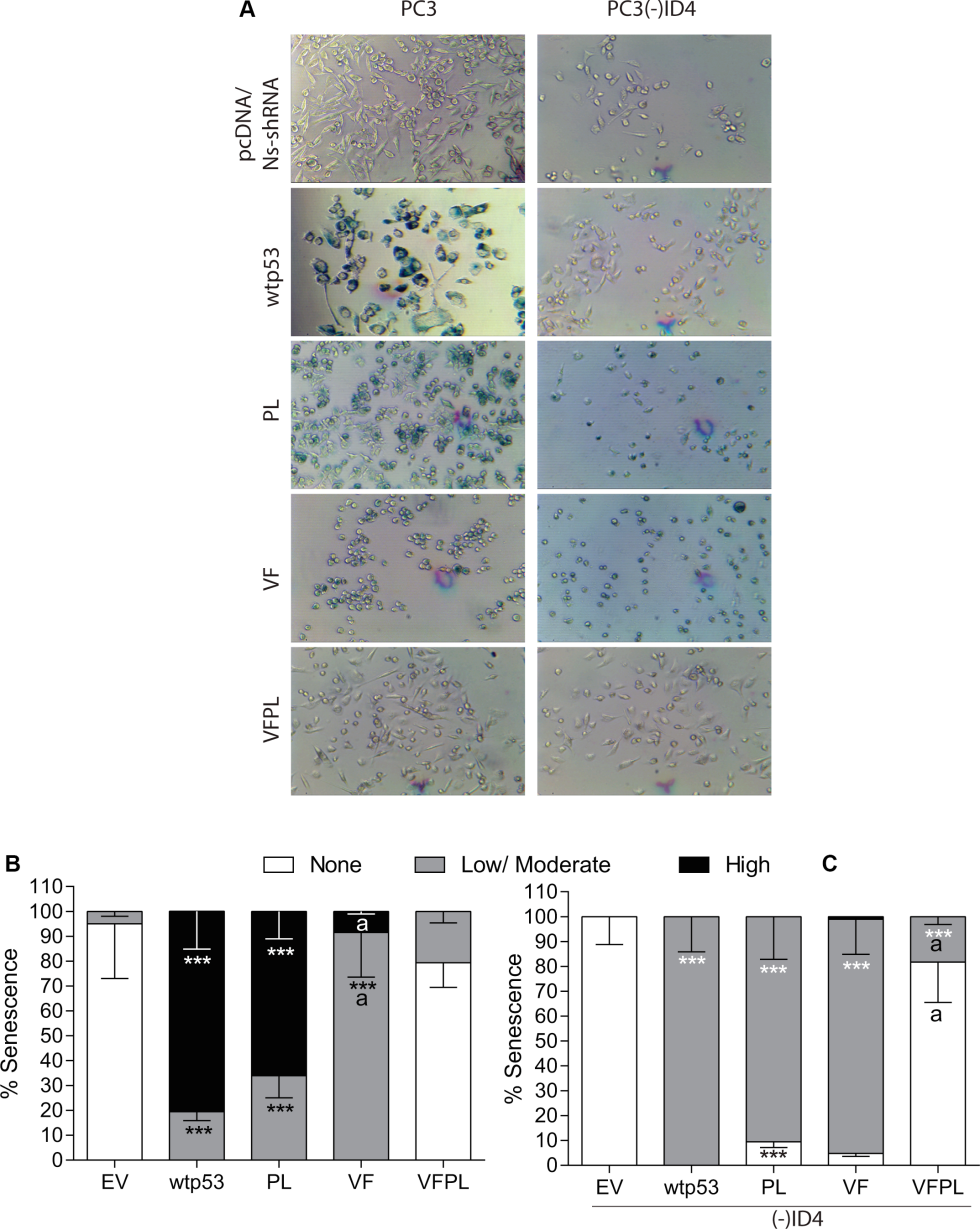
ID4 promotes p53-mediated senescence (**A**) ID4 promotes senescence in PC3 cells expressing EV, wild type, and mutant p53 compared to PC3 (-) ID4. Representative images shown. (**B**) Quantitative representation of senescence ranging from None, Low/moderate, and High in PC3 cells expressing EV, wild type, and mutant p53. (**C**) Quantitative representation of senescence ranging from None, Low/moderate, and High in PC3 (-) ID4 cells expressing EV, wild type, and mutant p53. ***:*P* < 0.001 in wild type and p53 mutants (p53 mutants: P223L and V274F) as compared to PC3 (EV); “a” (*P* < 0.001) in PC3 (-)ID4 with various p53 mutants as compared to PC3(-)ID4 cells transfected with wild type p53. Abbreviations: EV: Empty vector, wild type: Wild Type, PL: P223L, VF: V274F, VFPL: V274F+P223L.

The number of cells with strong (High) and those with light (Low-Moderate) blue staining were quantitated (Figure 3A). Over 60% of PC3 (wild type and mutant p53) cells stained positive for the SA-βgal and ~20% of those cells appeared to have flattened morphology (Figure 3A and 3B), indicative of senescence in PC3 (wild type and mutant p53) cells. The results summarized in (Figure 3B) demonstrated a significant increase in the number of cells with senescence associated beta-galactosidase (SA-βgal) staining in PC3 cells transfected with wild type p53 (High: 81%, Low-Moderate 19.5%) as compared to EV (High: None, Low-Moderate: 5%, *P* < 0.001). A varying degree in SA-βgal staining was also observed in the PC3+PL/ VF or VFPL. Interestingly, senescence was not significantly different in PC3+PL but a disproportionate increase in cells with low-moderate senescence was observed in VF cells (91.6%, *p* < 0.001) with a corresponding decrease in cells with high senescence (8.42, *p* < 0.001) as compared to PC3+wild type p53 cells. The senescence in VFPL cells was not significantly different as compared to the PC3 cells transfected with EV (Figure 3B).

A significant increase in senescent cells was also observed in PC3(-)ID4 cells transfected with either wild type or mutant p53. Interestingly cells with high senescence were not observed in PC3(-)ID4+wild type p53 cells but almost all cells showed low/moderate senescence. This is in stark contrast to PC3+wild type p53 cells in which a significant fraction of cells was highly senescent. The PL or VF mutants in PC3(-)ID4 also demonstrated low/ moderate senescence that was not significantly different from PC3(-)ID4+wild type p53 (Figure 3C). A decrease in the fraction of highly senescent PC3(-)ID4 cells suggests that loss of ID4 expression down-regulates senescence at a higher frequency than PC3 cells (Figure 3)

### The p53 hotspot mutants promotes apoptosis in an ID4 dependent manner

**Figure 4:**
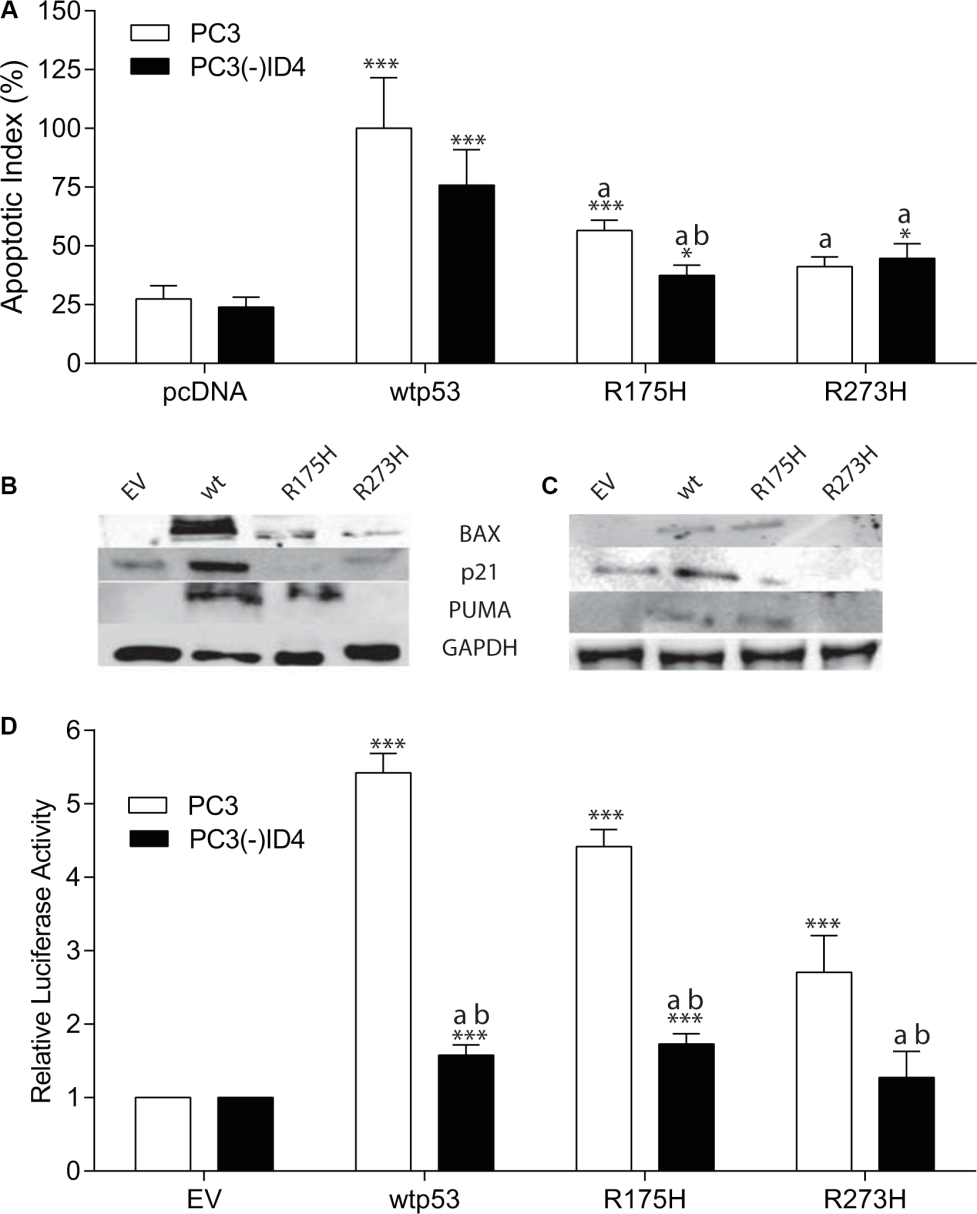
The p53 Hotspot mutants promotes apoptosis in an ID4 dependent manner (**A**, **B**) Protein expression profile of p21, BAX, and PUMA in PC3 and (**C**) PC3 (-) ID4 cells expressing EV, wild type, and hotspot mutant p53. Representative western blots of three different experiments are shown. (**D**) p53RE- luciferase reporter assay. A significant increase in apoptosis (***:*P* < 0.001, *:*p* < 0.05) in wild type and p53 mutants (p53 mutants: P223L and V274F) as compared to PC3 (EV); “a” (*P* < 0.001) in PC3 (-)ID4 with various p53 mutants as compared to PC3(-)ID4 cells transfected with wild type p53 and “b” (*P* < 0.05) is the statistical difference between PC3 and PC3(-)ID4 cells transfected with the same wild type or mutant p53 plasmid. Abbreviations: EV: Empty vector, wild type: Wild Type, PL: P223L, VF: V274F, VFPL: V274F+P223L.

p53 hotspot mutants are known to not only lose their tumor-suppressor function but also acquire oncogenic gain of function (GOF) [30]. Studies on hotspot mutant p53 knock-in models have shown manifestation of GOF by broader tumor spectrum and more metastasis compared with non-hotpot p53 mutants and the p53-null allele, which have largely been the focus of this study [7, 25, 31]. We therefore examined whether ID4 would have a similar effect on hotspot p53 mutants as well. We investigated the rate of apoptosis in the presence of p53 hotspot mutations (R175H and R273H) in PC3 cells (Figure 4A). Surprisingly, the R175H mutant was able to induce apoptosis (apoptotic Index 56.6%, *p* < 0.001) as compared to vector only control (27.5%) but was significantly lower (*p* < 0.001) as compared to PC3+wild type p53 (apoptotic index 100%). The apoptotic index of the R273H mutant (41%) was not significantly different from the vector only control.

Interestingly, a significant decrease in apoptosis was observed in PC3(-)ID4 cells with R175H mutant (37.5%, *P* < 0.01) as compared to the corresponding PC3 cells with the same mutant (56.6%) and PC3(-)ID4 cells with wild type p53 (75.8%, *p* < 0.001). However there was not a significant decrease in apoptosis between PC3 and PC3(-)ID4 cells with R273H mutant probably due to R273H classified as a DNA contact mutant.

The expression of p53 dependent apoptosis mediators is also consistent with the lower apoptotic index of R175H and R273H specifically. The expression of BAX in PC3 cells was in the following order p53 >> R175H > R273, whereas PUMA and p21 were not detected in PC3 cells with R273H (Figure 4B). In PC3(-)ID4 cells, the expression of all three p53 regulated genes was lower that their corresponding counterparts in PC3 cells however, BAX, PUMA and p21 was undetectable in in PC3(-)ID4+R273H (Figure 4C). These findings strongly suggested that ID4 can re-activate the function of well-established p53 mutations.

### ID4 promotes DNA binding and transcriptional activity of p53 hotspot mutants

We next investigated whether ID4 promotes DNA binding of the hotspot mutants in a p53 response element driven luciferase reporter assay. As shown in Figure 4D, the relative luciferase activity of R175H and R273H mutants in PC3 cells was significantly higher as compared to the empty vector control. The relative luciferase activity of R173H was significantly higher as compared to R273H (*P* < 0.001) whereas the relative luciferase activity of R173H (*p* < 0.01) and R273H (*p* < 0.001) was lower as compared to the relative luciferase activity of the wild type p53 (used as a positive control).

In PC3(-)ID4 cells, the relative luciferase activity of wild type (*p* < 0.001), R173H (*P* < 0.001) and R273H (*P* < 0.001) was significantly lower as compared to relative luciferase activity observed for the wild type p53 in PC3 cells. Furthermore the relative luciferase activity of R173H and R273H in PC3(-)ID4 was significantly lower (*p* < 0.001) as compared to the respective mutants in PC3 cells (Figure 4D). The mutant p53 luciferase plasmid (mt-p53RE) used as a negative control, as expected, did not result in luciferase activity (data not shown). These results clearly demonstrated that ID4 regulates the transcriptional activity of the p53 hotspot mutants also.

### ID4 promotes acetylation of p53

**Figure 5:**
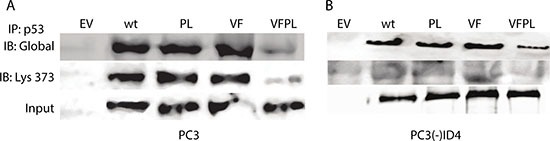
Acetylation of p53 (**A**) p53 immuno-precipitated from PC3 and (B) PC3(-) ID4 was blotted with antibodies against acetylated lysine (global) and p53 acetylated at K373 (Ac-320). An increase in interaction was observed in wild type and p53 mutants as compared to PC3 (EV) and decrease in interaction in PC3 (-) ID4 was observed in wild type and p53 mutants as compared to PC3(-) ID4 cells.

To investigate the mechanism by which ID4 may promote apoptosis and senescence, we explored the acetylation pattern on p53 in both PC3 and PC3(-)ID4 cell lines (Figure 5). The total p53 protein was first immuno-precipitated and then immuno-blotted with antibodies specific against global p53 acetylation and K373 acetylation only. Our results demonstrated increased global p53 lysine acetylation as well as increased acetylation of K373 in PC3 cells (Figure 5A). Whereas we observed a significant decrease in both global and site specific (K373) acetylation of p53 in PC3 (-) ID4 cells (Figure 5B). However, we were unable to observe significant global or site-specific acetylation (K373) in PC3 cells regardless of ID4 status with mutant p53-VFPL co-expressed compared to wild type and mutant p53 (V274F and P223L) expressed individually (Figure 5). This is likely due to co-expression of both mutants, which is in line with previous studies which reported that upon co-expression, both mutants together lose capability to perform p53-mediated processes [32].

### ID4 differentially modulates specific acetylation sites of p53

**Figure 6:**
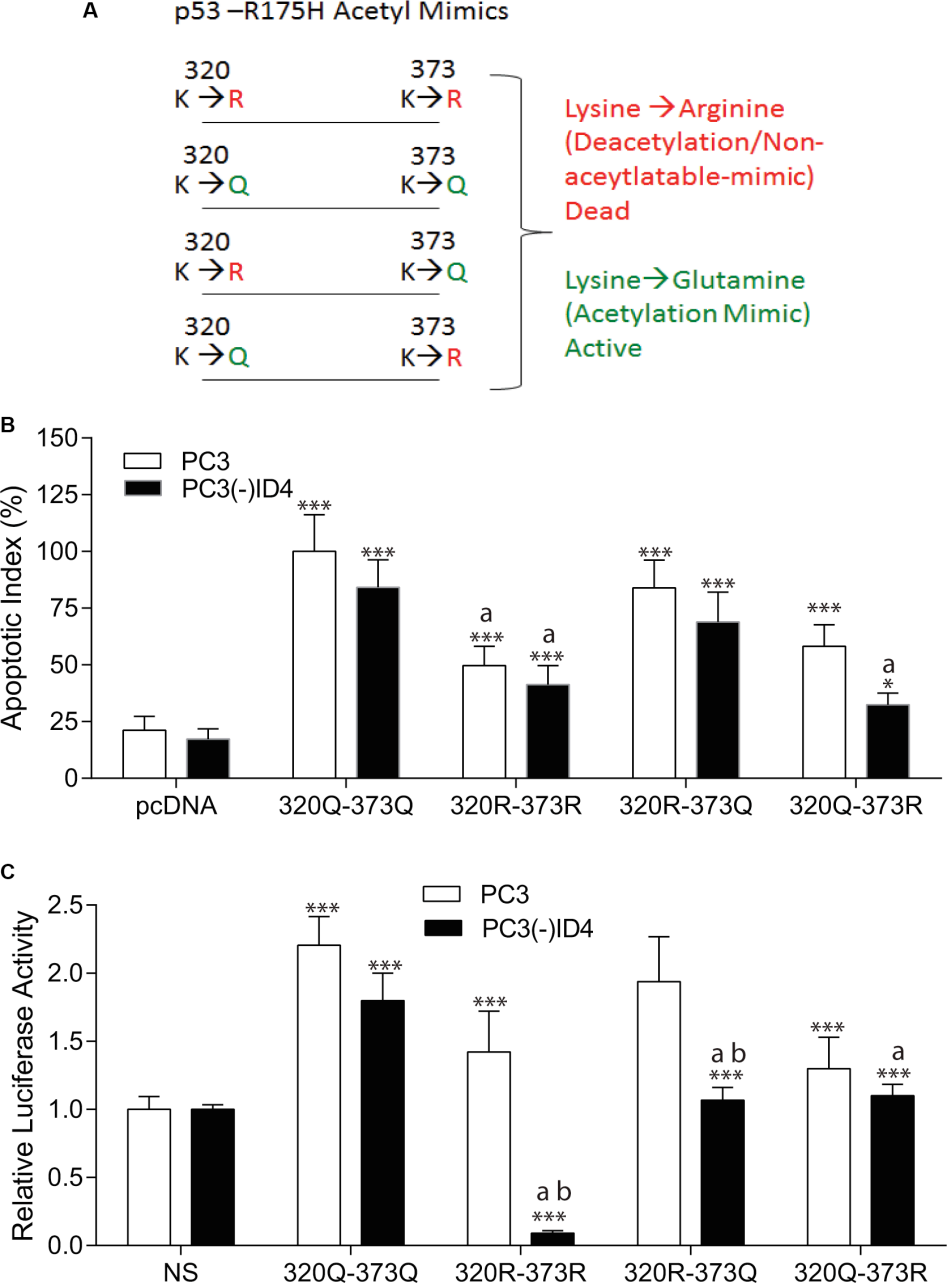
Schematic of p53-R175H Acetyl Mimics. (**B**) Comparative apoptotic index of acetylation mimics 320Q and 373Q on p53-R175H backbone in PC3 and PC3 (-) ID4 show significant decrease in apoptosis of 320Q-373R compared to 320Q-373R. ***:*P* < 0.001: between empty vector (EV) and acetylation mimics , wild type “a”: PC3 (-)ID4 and “b” (*p < 0.05) within the same transfectant between PC3 and PC3 (-)ID4 (**C**) Comparative p53RE-Lucifease reporter assay showed significant decrease in promoter binding in 320Q-373R as compared to 320Q-373Q on the p53-R175H backbone in PC3 cells. (***:*P* < 0.001) was observed in wild type and p53 mutants as compared to PC3 (EV) and “a”: PC3 (-)ID4 and “b” (**< 0.05) within the same transfectant between PC3 and PC3 (-)ID4.

Next, we investigated the specific acetylation of p53 within its C-terminal region that is known to regulate its stability and transcriptional activity, thereby facilitating active transcription of cell-cycle arrest, survival, or apoptotic gene programs [33]. However, the effects of acetylation and deacetylation on p53 activity seem to be cell-type dependent, and little is known about their consequence in epithelial cells of the prostate. Based on our earlier results that demonstrated ID4 dependent acetylation of K320 and K373 [28], we used an acetyl-mimic model to study the role of ID4 on site-specific residues critical to the regulation of p53 activity by using the acetylation and non-acetylation mimics (acetylation mimics: K320Q and K373Q and non-acetylation mimics: K320R and K373R) (Figure 6A). By overexpressing a collection of p53-R175H acetylation-mimic mutants in PC3 cells, our results suggest that specific acetylation at K373, and to a lesser extent at K320, are sufficient for inducing p53 apoptosis (Figure 6B) and restoration of DNA-binding capabilities (Figure 6C). Interestingly, the results suggested the acetylation mimics (320Q and 373Q) on R173H backbone promoted apoptosis irrespective of ID4 expression (Figure 4A, 100% in PC3 and 84% in PC3(-)ID4). On the contrary, apoptosis was significantly reduced in the presence of the non-acetylation mimics (320R and 373R) in the presence (PC3: 49%) or absence of ID4 (41%, PC3(-)ID4) (Figure 6B). The R175H with the 320Q and 373Q acetylation mimics also demonstrated transactivation potential as determined by their relative luciferase activity. The non-acetylation mimics (320R and 373R) also demonstrated transactivation potential in PC3 cells but not in PC3(-)ID4. But, in the absence of ID4, the lack of acetylation of flanking lysine could have resulted in the loss of activity of 320R and 373R in PC3(-)ID4. We then systematically evaluated the contribution or each acetylation site to understand their role on regulation the transactivation potential of R175H mutant in the absence of ID4. Collectively, the results suggest that the apoptosis in PC3(-)ID4 cells with 320Q and 373R mutant was significantly lower as compared to acetylation mimics. Furthermore the DNA binding activities with either of the K320 or K373 non-acetylation mutants was significantly lower in PC3(-)ID4 cells as compared to their activity in PC3 cells (Figure 6C). The results clearly suggested that these two acetylation sites may have an equal role on ID4 dependent re-activation of the R175H p53.

## DISCUSSION

Current understanding of the complex biological processes regulated by the p53 tumor suppressor is still not clear [33, 34]. A number of studies in recent years have focused on the design of novel anticancer drugs that can re-activate p53 mutants through various mechanisms [1, 35]. These studies raise the question of whether intracellular signals or pathways acting in a fashion similar to artificial compounds exist that can prevent mutant p53 loss of function [1]. In this study, we provide strong evidence that mutant p53 (DU145 specific mutants P223L and V274F and hotspot p53 mutants R175H and R273H) gains DNA binding and trans-activation potential largely through ID4-dependent acetylation.

Previous reports have characterized p53 mutations in prostate cancer cell lines DU145 and PC3 [32, 36, 37]. DU145 cells harbor two missense mutations on both alleles (V274F and P223L) and PC3 is rendered null for p53 due to a frame-shift deletion [32]. The mutant form of p53 studied in the DU145 cell line shows activation of mutant forms of p53 due to a temperature sensitive allele. Flow cytometry studies found induced expression of P223L at 32C, but not at 37C, and was as active as wild type p53 in the inhibition of colony growth. The V274F allele had no growth-inhibitory effect suggesting that the P223L allele mediates the tumor suppressor phenotype. Moreover, the V274F allele did not promote reporter gene activity of p21 or HDM2, whereas the P223L did induce both respective p53 target genes expression, providing further evidence that the P223L allele may regain wild type p53 tumor suppressor phenotype possibly due to a change in structural conformation at lower temperature. These studies support the larger role for non-artificial intervention in restoring mutant p53 due to inherent conformational flexibility in p53. Taken together, these findings [37] and our previous studies [28] led us to continue our investigation of ID4 dependent acetylation on DU145 specific p53 mutants as well as hotspot p53 mutations in p53-defecient cells of the prostate.

Our studies suggest that the p53-mediated processes of V274F to be similar to that of the hotspot p53 mutants (R175H and R273H), suggesting that V274F more likely shares properties of “real” cancer-derived mutants as shown by Gurova *et al*. [32]. The V274F allele induced less apoptosis, senescence, and induction of p53 downstream targets compared to the P223L allele. Both P223L and V274F when expressed in PC3 cells, showed some properties that are unusual for tumor-derived mutants, as they both could be stabilized by DNA damage, suppress growth, and did not possess dominant–negative gain-of-function activity, although, both mutants didn't share the same rate of induction of these processes compared to one another, complimentary to both previous studies [32, 37]. Importantly, it was also shown when both relatively weak mutants were co-expressed in one cell they kept a predominantly denatured conformation, accumulated in large amounts in the cell, didn't not show any increase after genotoxic stress, had no toxicity to any cells tested, and lacked transactivation function [32]. These findings align well with our results that show increased apoptosis and senescence with both relatively weak mutations individually. However, when both weak p53 mutants were co-expressed, we observed down regulation of p53-depedent processes supporting previous studies [28, 32].

In our earlier studies, we proposed that ID4 dependent activation of mutant p53 in DU145 cells is mediated via acetyl-transferases such as CBP/p300 or by other post-translational modifications that occur in an acetylation dependent manner. It has been well established that several C-terminal lysines (evolutionarily conserved across species, [38]) of p53 (K370, K372, K373, K381, K382) are acetylated by CPB/p300 [39]. Acetylation of residues found in the C-terminal of p53 promotes an open conformation by promoting the ability of its C-terminus to bind and occlude the DNA binding domain, thereby activating and enhancing p53 transcriptional activity [39, 38].

Studies have shown that acetylation of p53 is usually activating but may also be repressive, that is largely cell type dependent [40, 41] . In human cancer cell lines, the p300-mediated acetylation of p53 is essential for p21 expression and cell cycle arrest [38] but not in mouse embryo fibroblasts (MEFs) lacking CBP/p300 [40].

Our results show ID4 dependent acetylation of p53 as activating, consequently the DU145 and hotspot p53 mutants transfected into PC3 gains trans-activation potential and DNA binding activity as compared to PC3 cells that lack ID4. The gain of transactivation potential of mutant p53 in PC3 cells that express ID4 is supported by the observations that show increased p53 dependent luciferase reporter activity, direct DNA binding of mutant p53 and up-regulation of p53 target genes such as p21, BAX, and PUMA. Interestingly, BAX and in some cases p21 is not activated by most rumor derived p53 mutants [42]. However, our results suggest the p53 mutants overexpressed in PC3 cells expressing ID4 are capable of trans-activating not only p21, but BAX and PUMA as well. In contrast, PC3 cells that lack ID4, demonstrated decreased regulation of all aforementioned downstream targets and DNA-binding. These results strongly suggest that ID4 has a role in facilitating both DNA binding as well as trans-activation potential of mutant p53. Lastly, the ID4 dependent post-translation modifications within p53 (discussed below) provide a molecular basis for p53-mediated modulation of transcriptional activity.

To elucidate the mechanism by which ID4 promotes p53-mediated transcriptional activity in PC3 cells we focused on ID4 dependent acetylation of p53. The results suggested an increased global p53 lysine and site-specific lysine (K373) acetylation in wild type and mutant p53 in an ID4 dependent manner, largely supporting our earlier studies in DU145 cells [28]. Moreover, we found no significant acetylation of mutant p53 (VFPL) when co-expressed, regardless of ID4 status as compared to wild type and mutant p53 (V274F and P223L) expressed individually. Our data therefore is consistent with previous findings showing minimal trans-activation potential of both mutants when co-expressed [32, 37].

Acetyl-mimics were used as a model to study the effects of p53 acetylation and de-acylation (acetyl-mimics: K320Q/R and K373Q/R) by studying of site-specific residues critical to the regulation of p53 activity. By overexpressing a collection of acetylation-mimic on the p53-R175H backbone mutants in PC3 cells, we show that specific acetylation at K373, and to a lesser extent at K320, are sufficient for inducing p53 target gene dependent transactivation, apoptosis and restoration of DNA-binding capabilities in an ID4 dependent manner providing further evidence on the role of ID4 in modulating acetylation of p53. Interestingly, the inhibitory effect of deacetylation/non-acetylation mimics (373R and 320R) on p53 induced apoptosis and DNA-binding capability seemingly disrupts the global transcriptional program of p53 by decreasing these activities. Moreover, ID4 may be preferentially modulating apoptosis and DNA-binding capabilities at higher rate via acetyl-mimic K373Q as compared to K320Q suggesting the K373 is critical for ID4 dependent p53 acetylation. Although not significant we did observe lower relative luciferase activity and apoptosis in K320R and K373R (deacytlation mimics) as compared to K320Q and K373Q acetyl-mimics in PC3 cells. Interestingly, Liu *et al*. [43] found similar results when they reported activation of downstream target a p21-thymidine kinase construct was modest (15%) with deacytlation mimics; this result might have been due to the presence of two flanking lysines (K319 and K321), one of which might be an optional binding site for PCAF [8], which could also provide an explanation of apoptotic activity and luciferase activity observed in our model with both K320R and K373R deacetylation mimics.

Apoptosis and to a lesser extent cell cycle arrest and senescence are largely regulated by acetylated p53 [44]. Several p53 null cell lines including PC3, upon treatment with HDAC inhibitors that block the de-acetylation of p53 show induction of p21, but not BAX indicating a distinct role for acetylation dependent cell cycle arrest and senescence [45]. Studies indicating a role for acetylation dependent cell cycle arrest and senescence independent of p53 add an interesting and significant point of discussion to our study. It is plausible that ID4 mediated acetylation alone, can promote cell cycle arrest and senescence independent of p53, suggesting different ID4 dependent acetylation pathways. Moreover, recognition of p53-independent processes that effect cell cycle arrest and senescence provide a basis for future exploration into the possibility of ID4 playing a larger role as a regulator of acetylation. Studies have also shown that stabilization of p53 at lysine 373, induces cell cycle arrest and apoptosis in LNCaP prostate cancer cells, while TSA an HDAC inhibitor stabilizes the acetylation of p53 at lysine 382 induces only cell cycle arrest in the cell line [12, 44]. Acetylation dependent cell fate determination in context of apoptosis and senescence via p53-mediated processes would be a promising study as possible mechanisms that lead to p53 directed cellular death or senescence are not well understood processes.

Cell type differences also influence the role of acetylation in apoptosis. In HCT116 colorectal cells, loss of CBP/p300 dependent p53 acetylation leads to increased expression of PUMA and apoptosis following DNA damage, [46]. The acetylation at K381 and K382 in neuronal cells also inhibits p53 binding to the PUMA promoter, preventing PUMA expression and DNA damage-induced cell death [47]. These results suggest that CBP/p300 expression normally suppresses p53-dependent apoptosis in neurons and HCT116 cancer cells.

As noted, studies have shown that the p53 mutations are indeed not all functionally equivalent, hence reports of p53 mutants with differential effects on the cell [9]. In the context of our study, p53 mutants in PC3 cells that express ID4 are able to promote p21, BAX, and PUMA expression. At least two of the p53 hotspot mutants R175H and R273H, which are well known cancer-derived gain-of-function p53 mutants accounting for 15% of all p53 mutations in cancer and have been shown to have crippling effects on knock-in mouse models [8, 48, 49]. Taken together, this study supports exploring a profile of other known p53 hotspot mutants and study of their inherent structural flexibility in context of acetylation.

In conclusion, our present study finds that ID4 plays a pivotal role in modulation of acetylation and preferentially acts to acetylate K373 for transactivation of p53 in context of both DU145 specific p53 mutants and hotspot mutants overexpressed in PC3 cells. This phenomenon provides clear evidence that ID4 may serve as a physiological agent to restore p53 wild type function to various mutant forms of p53 in a non-cell line/non-mutation specific manner. While this data is intriguing, it is of great interest to determine if this phenomenon of p53 restoration via ID4 is effective in other organ systems (outside prostate cancer) as well as with a variety of other p53 mutations. Continued studies elucidating the mechanism of action will help to unravel the complex biological processes of ID4 and p53 in cancer. The strong anti-cancer effect of ID4 previously reported in prostate cancer and cross-talk with p53 will provide a strong framework of study in other systems, which eventually could serve as an attractive therapeutic approach [50]. ID4 promotes acetylation possibly by recruiting CBP/P300 type acetyl transferases however whether other acetyltransferases can also be recruited by ID4 independent of p53 for other proteins remains to be investigated. In the future, the studies demonstrating the effect of ID4 as a tumor suppressor in which certain acetyl transferases have been silenced (in the absence or presence of wild type or mutated p53) may help to unravel the complex mechanism of action of ID4.

## MATERIALS AND METHODS

### ID4 silencing in prostate cancer (PCa) cell line

PC3 prostate cancer cell line was purchased from ATCC and cultured as per ATCC recommendations. ID4 was stably silenced in PC3 cells using gene specific shRNA retroviral vector as described earlier [28].

Mutagenesis of p53: The retroviral p53 vector previously described [28] was manipulated via mutation induced by site-directed mutagenesis mimicking DU145 cells that harbor mutant p53 (P223L and V274F) [51] via QuickChange II Site-Directed Mutagenesis kit (Agilent Technologies, USA). Hotspot mutations of p53 (R273H and R175H) [52] were purchased from Addgene, Inc: pCMV-Neo-Bam p53 R175H (Addgene plasmid #16436) and pCMV-Neo-Bam p53 R273H (Addgene plasmid # 16439) [53] was a gift from Bert Vogelstein. The following primers were used for generating the P223L and V274F mutants:

### Primers for P223L mutant

Forward: 5′-CTA TGA GCC GCC TGA GGT TGG CTC TG

Reverse: 5′-CAG AGC CAA CCT CAA GCG CAT AG

Primers for V274F mutant:

Forward: 5′ AGG TGC GTG TTT GTG CCT GTC CTG G

Reverse: 5′ CCA GGA CAG GCA CAA AAA CGC ACC T

The following primer pair was used for reverse transcriptase polymerase chain reaction (RT-PCR) and sequencing of p53 for measuring expression levels and verifying the mutants by sequencing the PCR product:

P53 wild type

Forward 5′-GCT CAG ATA GCG ATG GTC TG

Reverse: 5′ TCT TCT TTG GCT GGG GAG AG

Western Blot Analysis: 30 ug of total protein, extracted from cultured prostate cancer cell lines using M-PER (Thermo Scientific) was size fractionated on 4–20% SDS-polyacrylamide gel. The fractionated proteins were transferred onto a nitrocellulose membrane (Whatman) and subjected to western blot analysis using respective protein specific antibodies. After washing with 1x PBS, 0.5% Tween 20, the membranes were incubated with horseradish peroxidase (*HRP*) coupled secondary antibody against rabbit IgG and visualized using the Super Signal West Dura Extended Duration Substrate (Thermo Scientific) on Fuji Film LAS-3000 Imager.

### Apoptosis assay

Apoptosis was quantified using Propidium Iodide and Alexa Fluor 488 conjugated Annexin V (Molecular Probes) as described previously [54].

### Senescence associated (SA)-β-galactosidase assay

The senescence associated-β-galactosidase assay was performed essentially as described earlier [55]. Briefly, the cells were cultured in six well plates with respective media. The cells at 60–70% confluency were stained for senescence associated-β-galactosidase (SA-βgal) staining kit (Cell signaling) as per manufacturer's instructions. At least 15 representative fields were randomly selected for the quantitation of the percentage of SA-βgal positive cells. The images were captured in both phase contrast and bright field to better visualize cellular details.

### p53 Activity Assay

p53 DNA binding activity and quantitation on nuclear extracts was performed as described previously [28].

### Transient transfections and reporter gene assay

PC3 cells cultured in 96-well plates (70–80% confluency) were transiently transfected with reporter plasmids by FuGENE HD transfection reagent (Promega) as described earlier [28]. Briefly, the PG13-luc (containing 13 copies of wild type p53 binding sites [26], Addgene) or MG15-luc (containing 15 mutant p53 binding sites [26], Addgene) with pGL4.74 (hRluc/TK: Renilla luciferase, Promega) plasmid DNA was transfected in a 10:1 ratio. After 24 h, the cells were assayed for Firefly and Renilla luciferase activities using the Dual- Glo Luciferase reporter assay system (Promega) in LUMIstar OPTIMA (MHG Labtech). The results were normalized for the internal Renilla luciferase as control.

### Co-immunoprecipitation

Co-immunoprecipitations were performed to detect the protein-protein interactions. Briefly, protein specific IgG (anti-p53 or –ID4) was first immobilized to Protein A Mag beads by incubating overnight at 4C as per manufacturer's instructions (Protein A Mag beads, GenScript). The co-elution of IgG following immuno-precipitation was minimized by cross linking, IgG on protein A mag beads as described in the kit. To detect protein interactions, the cross-linked protein specific IgG-protein A-Mag beads were incubated overnight (4C) with freshly extracted total cellular proteins (500 μg/ml). The complex was then eluted with 0.1M Glycine buffer (pH 2–3) after appropriate washing with PBS and neutralized by adding neutralization buffer (1M Tris, pH 8.5).

### Generation of acetyl-p53 mimics constructs

Plasmids containing sequences for human p53-R175H DNA-binding mutants: K320R+K373R, K320Q+K373Q, K320R+K373Q, and K320Q+K373R were commercially synthesized (GenScript). The following primers were used:

Lysine 373 Non-Acetylation Mimic

Forward: K373R 5′-CAC CTG AAG TCC AAA AGG GGT CAG TCT ACC TCC

Reverse: K373R 5′-GGA GGT AGA CTG ACC CCT TTT GGA CTT CAG GTG

Lysine 373 Acetylation Mimic

Forward: K373Q 5′-CAC CTG AAG TCC AAA GAG GGT CAG TCT ACC TCC

Reverse: K373Q 5′-GGA GGT AGA CTG ACC CTC TTT GGA CTT CAG GTG

Lysine 320 Non-Acetylation Mimic

Forward: K320R 5′-TCT CCC CAG CCA AAG AGG AAA CCA CTG GAT GGA

Reverse: K320R 5′-TCC ATC CAG TGG TTT CCT CTT TGG CTG GGG AGA

Lysine 320 Acetylation Mimic

Forward: K320Q 5′-TCT CCC CAG CCA AAG GAG AAA CCA CTG GAT GGA

Reverse: K320Q 5′-TCC ATC CAG TGG TTT CTC CTT TGG CTG GGG AGA

### Statistical analysis

Data was analyzed by SPSS 13.0 statistics software. Experimental data is presented as means ± standard error of the mean (SEM). A *p*-value of < 0.05 was considered statistically significant.
